# Monoterpene Hydroxy Lactones Isolated from *Thalassiosira* sp. Microalga and Their Antibacterial and Antioxidant Activities

**DOI:** 10.3390/molecules29215175

**Published:** 2024-10-31

**Authors:** Alcina M. M. B. Morais, Decha Kumla, Valter F. R. Martins, Ana Alves, Luis Gales, Artur M. S. Silva, Paulo M. Costa, Sharad Mistry, Anake Kijjoa, Rui M. S. C. Morais

**Affiliations:** 1 CBQF—Centro de Biotecnologia e Química Fina—Laboratório Associado, Escola Superior de Biotecnologia, Universidade Católica Portuguesa, Rua Diogo Botelho 1327, 4169-005 Porto, Portugal; abmorais@ucp.pt (A.M.M.B.M.); decha.ku@go.buu.ac.th (D.K.); s-vfrmartins@ucp.pt (V.F.R.M.); anajoao93@hotmail.com (A.A.); 2 ICBAS—Instituto de Ciências Biomédicas Abel Salazar, Universidade do Porto, Rua de Jorge Viterbo Ferreira, 228, 4050-313 Porto, Portugal; lgales@ibmc.up.pt (L.G.); pmcosta@icbas.up.pt (P.M.C.); ankijjoa@icbas.up.pt (A.K.); 3 Instituto de Biologia Molecular e Celular (i3S-IBMC), Universidade do Porto, Rua de Jorge Viterbo Ferreira, 228, 4050-313 Porto, Portugal; 4 Departamento de Química & QOPNA, Universidade de Aveiro, 3810-193 Aveiro, Portugal; artur.silva@ua.pt; 5 Department of Chemistry, University of Leicester, University Road, Leicester LE 7 RH, UK; scm11@leicester.ac.uk; 6 Interdisciplinary Centre of Marine and Environmental Research (CIIMAR), Terminal de Cruzeiros do Porto de Leixões, Av. General Norton de Matos s/n, 4450-208 Matosinhos, Portugal

**Keywords:** *Thalassiosira* sp., microalgae, monoterpenoid lactones, loliolide, *epi*-loliolide, antibacterial activity, antioxidant activity

## Abstract

Two monoterpenoid lactones, loliolide (**1**) and *epi*-loliolide (**2**), were isolated from the crude dichloromethane extract of a microalga, *Thalassiosira* sp.). The structures of loliolide (**1**) and *epi*-loliolide (**2**) were elucidated by 1D and 2D NMR analysis, as well as a comparison of their ^1^H or/and ^13^C NMR data with those reported in the literature. In the case of loliolide (**1**), the absolute configurations of its stereogenic carbons were confirmed by X-ray analysis, whereas those of *epi*-loliolide (**2**) were determined by NOESY correlations. Loliolide (**1**) and *epi*-loliolide (**2**) were tested for their growth inhibitory activity against two Gram-positive (*Staphylococcus aureus* ATCC 29213, *Enterococcus faecalis* ATCC 29212) and two Gram-negative (*Escherichia coli* ATCC 25922, *Pseudomonas aeruginosa* ATCC 27853) bacteria, as well as one clinical isolate (*E. coli* SA/2, an extended-spectrum β-lactamase producer-ESBL) and two environmental isolates, *S. aureus* 74/24, a methicillin-resistant (MRSA), and *E. faecalis* B3/101, a vancomycin-resistant (VRE) isolates. The results showed that none of the tested compounds exhibited antibacterial activity at the highest concentrations tested (325 μM), and both revealed low antioxidant activity, with ORAC values of 2.786 ± 0.070 and 2.520 ± 0.319 µmol TE/100 mg for loliolide (**1**) and *epi*-loliolide (**2**), respectively.

## 1. Introduction

Microalgae are microorganisms that constitute a diverse group of microscopic prokaryotic (cyanobacteria) and eukaryotic photosynthetic organisms of paramount ecological importance [1]. While microalgae are mainly found in aquatic environments (49.78% in freshwater and 44.48% in seawater), some species also grow in contaminated and extreme environments, including thermal and glacial lakes [2,3]. Microalgae are rich sources of highly bioactive compounds, whose origin can be sourced directly from primary and secondary metabolisms such as carotenoids, polyunsaturated fatty acids (PUFAs), phycobiliproteins, polysaccharides and phycotoxins with high complexity and unlimited diversity of pharmacological and/or biological activities, including antioxidant [4], antiaging [5,6], antiviral [7], anticoagulant [8], anti-inflammatory [9], antimicrobial [10], anticancer [8,11], and antitumoral activities [12,13].

Microalgae may play a critical role in sustainability by contributing to several Sustainable Development Goals (SDGs) of the United Nations. In addition to being a renewable energy source (e.g., biofuel production), microalgae offer benefits in areas like carbon capture, bioremediation, food security, and waste treatment. They are also a source of new ingredients and bioactive compounds in the food/feed and pharmaceutical sectors. While microalgae are a promising tool for sustainability, challenges such as economic scalability and technological bottlenecks remain. Therefore, further research and development are required to fully assess their potential across the different applications [14]. The *Thalassiosira* sp. is a diatom genus known for its diverse range of species that plays crucial roles in marine ecosystems, including primary production and nutrient cycling. The genus *Thalassiosira* consists of a diverse group of photosynthetic eukaryotes that make up a vital part of marine and freshwater ecosystems. In addition, they are essential for carbon cycling because they convert carbon dioxide into organic matter through photosynthesis, supporting aquatic food webs and significantly contributing to global oxygen production. This genus has received increasing interest due to its rich nutritional value and high polyunsaturated fatty acids (PUFAs) contents, such as docosahexaenoic acid (DHA) and eicosapentaenoic acid (EPA), although pigments, such as fucoxanthin, peridinin, chlorophyll A, and chlorophyll C, as well as diadinoxanthin have been reported [15,16].

Loliolide, together with its less abundant stereoisomer *epi*-loliolide, has been isolated from different sources, including seaweeds and freshwater algae, showcasing a broad spectrum of applications. However, these compounds have not been isolated from *Thalassiosira* sp. Loliolide is a monoterpenoid lactone that has been widely studied for its diverse biological activities and structural properties. It was initially identified in *Lolium perenne* [17], and it has demonstrated significant antioxidant, anti-inflammatory, and anti-cancer properties [18,19]. Recent research highlights its potential in neuroprotection and as a therapeutic agent for neurological diseases [18]. Additionally, loliolide exhibits promising anti-aging properties, anti-melanogenic effects, and oxidative stress protection [19,20]. Furthermore, loliolide interaction with serotonin transporters suggests its potential in treating central nervous system disorders [21]. The compound has also been studied for its hepatocellular carcinoma activity, with recent findings indicating its efficacy in reducing cancer cell viability [22]. Overall, loliolide wide-ranging bioactivities and structural attributes underscore its significance in both ecological and therapeutic contexts.

The objective of the present study was to isolate and identify secondary metabolites from a microalga *Thalassiosira* sp. in the ongoing search for new natural antibiotics from microalgae.

## 2. Results and Discussion

Compounds **1** and **2** were elucidated by analysis of their 1D and 2D NMR spectra, HRMS data, and comparison of their spectral data to those reported in the literature.

**Figure 1:**
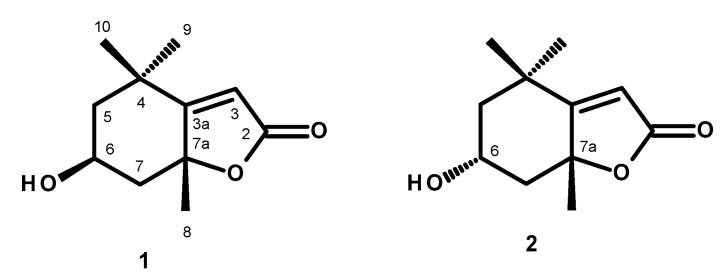
Structures of loliolide **(1)** and *epi*-loliolide **(2)**.

**Figure 2:**
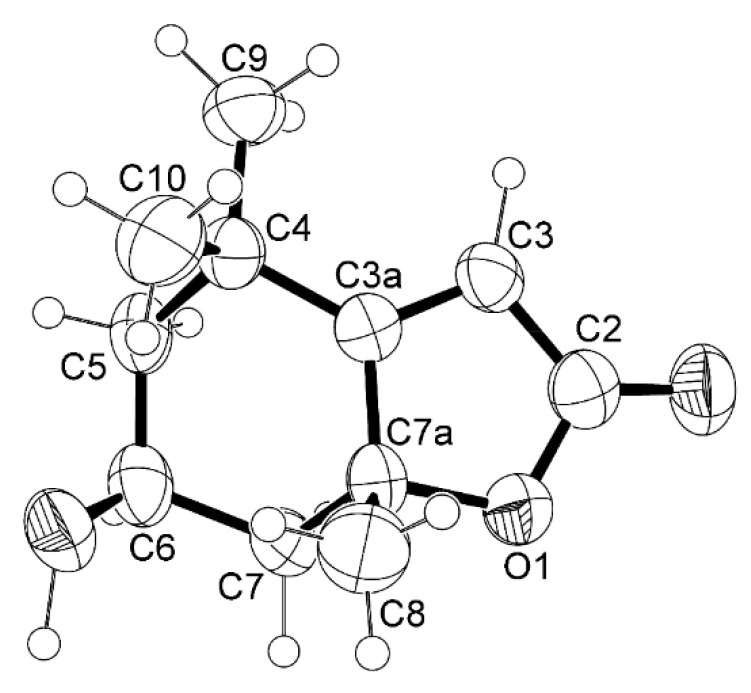
ORTEP diagram of loliolide (**1**).

Compound **1** was isolated as a white crystal, and its molecular formula C_11_H_16_O_3_ was established based on the (+)-HRESIMS *m*/*z* 197.1178 [M+H]^+^ (calc. for C_11_H_17_O_3_^+^, 197.11722), indicating four degrees of unsaturation. The ^1^H NMR spectrum of **1** (Table 1, Figure S1-1) displayed a vinylic proton at δ_H_ 5.69 (H-3, *s*) and two pairs of diastereotopic methylene protons at δ_H_ 2.00 (1H, *ddd*, *J* = 14.5, 2.7, 2.7 Hz, H-5_ax_) and δ_H_ 1.53 (1H, *dd*, *J* = 14.5, 3.7 Hz, H-5_eq_), δ_H_ 2.44 (1H, *ddd*, *J* = 14.0, 2.5, 2.5 Hz, H-7_ax_) and δ_H_ 1.77 (1H, *dd*, *J* = 14.0, 4.0 Hz, H-7eq) and three methyl singlets at δ_H_ 1.27 (H-9), 1.48 (H-10) and 1.79 (H-8). The ^13^C NMR spectrum (Table 1, Figure S1-2) displayed eleven carbon signals which, in combination with DEPT and HSQC spectra (Figure S1-3), can be categorized as a quaternary sp^2^ at δ_C_ 182.8, a ketone carbonyl sp^2^ at δ_C_ 172.2, a methine sp^2^ at δ_C_ 122.8, an oxymethine sp^3^ at δ_C_ 66.7, two quaternary sp^3^ (δ_C_ 36.0 and 87.0), two methylene sp^3^ (δ_C_ 45.6 and 47.2), and three methyl (δ_C_ 30.7, 27.0 and 26.5) carbons. Through the comparison of the ^1^H and ^13^C chemical shift values of the COSY (Figure S1-4) and HMBC (Figure S1-5) correlations with those reported in the literature [22,23], **1** was preliminarily identified as loliolide. The structure and the absolute stereochemistry (6*S*, 7a*R*) of loliolide (**1**) (Figure 1) were confirmed by X-ray analysis, and the ORTEP view (CCDC 2096018 and CCDC 2095981) is shown in Figure 2 and Figure S1-6.

Loliolide has been previously reported from a few macroalgal species, such as *Gracilaria lemaneiformis* [24], *Undaria pinnatifida* [25], and *Sargassum ringgoldianum* [26], and also from a marine microalga, *Tisochrysis lutea* [22]. Intriguingly, this compound has also been isolated from terrestrial plant species, namely *Salvia divinorum* [27], *Eucommia ulmoides* [28], and *Mondia whitei* [21]. However, this is the first report of the isolation of loliolide (**1**) from the microalga *Thalassiosira* sp.

**Figure 3:**
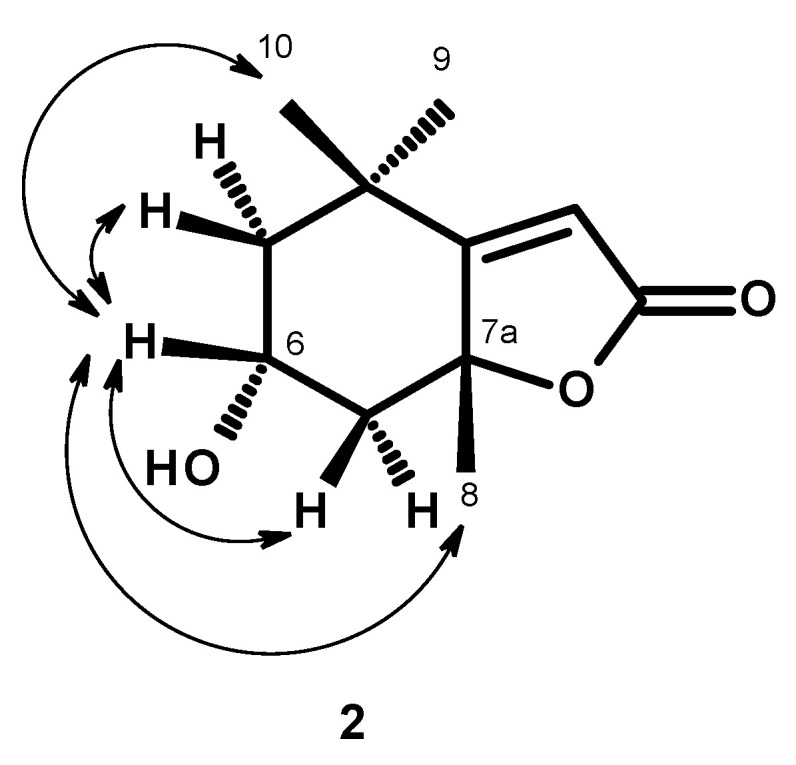
Key NOESY (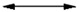) correlations in compound **2**.

Compound (**2**) was isolated as a white amorphous solid, [α]_D_^20′^ +80 (c 0.05, CHC1_3_), and its molecular formula was established as C_11_H_16_O_3_ by the (+)-HRESIMS *m/z* 197.1178 [M+H]^+^ (calc. for C_11_H_17_O_3_^+^, 197.11722), indicating four degrees of unsaturation. Thus, **2** is an isomer of **1**. The ^1^H NMR spectrum of **2** (Table 2, Figure S2-1) displayed a vinylic proton at δ_H_ 5.68 (H-3, *s*) and two pairs of diastereotopic methylene protons at δ_H_ 2.02 (1H, *ddd*, *J* = 12.8, 4.3, 2.3 Hz, H-5_ax_) and δ_H_ 1.31 (1H, *dd*, *J* = 12.2, 12.2 Hz, H-5_eq_), δ_H_ 2.51 (1H, *ddd*, *J* = 11.8, 4.0, 2.3 Hz, H-7_ax_) and δ_H_ 1.48 (1H, *dd*, *J* = 11.8, 11.8 Hz, H-7eq) and three methyl singlets at δ_H_ 1.24 (H-10), 1.29 (H-9), and 1.56 (H-8). The ^13^C NMR spectrum (Table 2, Figure S2-2) displayed eleven carbon signals which, in combination with DEPT and HSQC spectra, can be categorized as a quaternary sp^2^ at δ_C_ 181.3, a ketone carbonyl sp^2^ at δ_C_ 171.9, a methine sp^2^ at δ_C_ 113.0, a methine sp^3^ at δ_C_ 64.8, two quaternary sp^3^ (δ_C_ 35.1 and 86.8), two methylene sp^3^ (δ_C_ 47.8 and 49.7) and three methyl (δ_C_ 29.9, 25.5 and 25.0) carbons. The ^1^H and ^13^C NMR spectra of **2** were similar to those reported for *epi*-loliolide [17,18]. The relative configuration of C-6 was established as opposite to that of C-6 of **1** by NOESY correlations from H-6 to Me-8 and Me 10 (Figure 3). Since the absolute configuration at C-6 and C-7a of **1** were determined, the absolute configurations at C-6 and C-7a in 2 are established as 6*R*, 7a*R*, thus confirming the structure of **2** as *epi*-loliolide (Figure 1). The COSY, HSQC, HMBC and NOESY spectra are presented in Figures S2-3, S2-4, S2-5 and S2-6, respectively.

*epi*-Loliolide (**2**) was first isolated from terrestrial plants, *Viburnum dilatatum* Thunb (family Caprifoliaceae) [29] and *Excoecaria cochinchinensis* (family Euphorbiaceae) [30]. This compound has also been reported in brown macroalgae, *Undaria pinnatifida* [25], *Sargassum thunbergii* [31], *S. naozhouense* [32]. To the best of our knowledge, this is the first report of the presence of *epi*-loliolide in a diatom *Thalassiosira* sp.

Loliolide (**1**) and *epi*-loliolide (**2**) were tested for their antibacterial activity against Gram-positive and Gram-negative bacteria, and their minimum inhibitory concentrations (MIC) and the minimum bactericidal concentrations (MBC) for several reference strains and environmental multidrug-resistant isolates were determined. In the range of concentrations tested, none of the compounds was active against Gram-negative bacteria. Regarding the antibacterial activity against Gram-positive bacteria, **1** and **2** were ineffective against all Gram-positive strains tested, with MIC values higher than the highest concentrations tested, 325 μM (64 μg/mL), for the reference strains (*E. faecalis* ATCC 29212, *S. aureus* ATCC 29213) and the multidrug-resistant strains (*E. faecalis* (VRE) B3/101 and *S. aureus* (MRSA) 66/1) (Table 3). Compounds **1** and **2** were also investigated for their potential to prevent biofilm formation on all four reference strains by measuring the total biomass through the crystal violet assay. None of the compounds inhibited the biofilm formation of the bacteria tested. Regarding the screening for potential synergies between the test compounds and clinically relevant antibiotics on the multidrug-resistant isolates, by the disk diffusion method and checkerboard assay, none of the compounds revealed a synergistic association with antibiotics, as determined by the different methodologies used.

The antioxidant activity of loliolide was evaluated by three different assays: radical cation-based assay (ABTS), radical scavenging ability (DPPH), and oxygen radical absorbance capacity (ORAC). According to these three assays, loliolide did not present a remarkable antioxidant activity, with lower ABTS, DPPH and ORAC values than butylated hydroxytoluene (Table 4). *epi*-Loliolide did not reveal any activity using ABTS and DPPH methods. Using ORAC it revealed a similar activity as loliolide (not significantly different). Silva et al. [18] found similar ORAC results (24.22 ± 3.45 µmol TE/g) for loliolide isolated from *Codium tomentosum*.

The failure of loliolide to inhibit bacterial growth could be hypothesized to arise from a combination of its chemical stability [33], lack of specific binding interactions with bacterial targets [34], poor permeability into bacterial cells [35], and potential degradation by bacterial enzymes [34]. These factors, combined with the loliolide bioactivity profile [18], which might be more suited for anti-inflammatory or antioxidant actions, make it less likely to function effectively as an antibacterial compound [36].

Loliolide and *epi*-loliolide, two naturally occurring compounds, have exhibited various biological activities, each with different degrees of efficacy. However, in general, loliolide shows higher antioxidant [37], anti-inflammatory [38], antitumor [22], and neuroprotective bioactivities [18] than *epi*-loliolide. Structure-activity relationship (SAR) studies focus on how changes in the chemical structure impact the biological activity of monoterpenes and their derivatives [39,40]. In the case of loliolide, the relatively simple and rigid nature of the lactone structure might limit the number of structural modifications that could enhance biological activity. Nevertheless, various chemical modifications could be suggested to improve the biological activity of loliolide, focusing on altering its core structure or functional groups. Possible chemical modifications include: adding electron-donating groups (e.g., hydroxyl groups) to the structure [41] and increasing conjugation in the ring system [42] to improve the antioxidant properties of loliolide and *epi*-loliolide; modifying the lactone ring, such as adding bulky substituents, which could make the compounds more reactive towards inflammation-related enzymes [38]; attaching large hydrophobic groups (e.g., alkyl or aromatic groups) or, on the other hand, adding amine groups or other reactive functional groups that could improve interactions with bacterial and tumor cell membranes [33], therefore, enhancing antimicrobial and antitumor effects.

## 3. Materials and Methods

### 3.1. General Experimental Procedures

Melting points were determined on a Bock monoscope and were uncorrected. Optical rotations were measured on an ADP410 Polarimeter (Bellingham + Stanley Ltd., Tunbridge Wells, Kent, UK). ^1^H and ^13^C NMR spectra were recorded at ambient temperature on a Bruker AMC instrument (Bruker Biosciences Corporation, Billerica, MA, USA) operating at 300 and 75 MHz, respectively. High-resolution mass spectra were measured with a Waters Xevo QToF mass spectrometer (Waters Corporations, Milford, MA, USA) coupled to a Waters Aquity UPLC system. A Merck (Darmstadt, Germany) silica gel GF_254_ was used for preparative TLC, and a Merck SiO_2_ gel 60 (0.2–0.5 mm) was used for column chromatography.

### 3.2. Extract Preparation from Microalgae

The *Thalassiosira* sp. biomass (ref. A4FEXTCA_0018) was kindly donated by the Portuguese company A4F—Algae for Future. The microalgae belong to the culture collection of A4F. The dried microalga (500 g) was transferred into separate Erlenmeyer flasks (1000 mL). Then, 500 mL of dichloromethane (CH_2_Cl_2_) was added to each flask and stirred using a magnetic stirrer for 24 h and then filtered with Whatman No. 1 filter paper. The organic solutions were combined and evaporated under reduced pressure to give 29.0 g of the crude dichloromethane extract, which was applied on a column of silica gel (385 g) and eluted with mixtures of petrol-CHCl_3_ and CHCl_3_-Me_2_CO, wherein 250 mL fractions (Frs) were collected as follows: Frs 1–39 (petrol), 40–77 (petrol-CHCl_3_, 9:1), 78–104 (petrol-CHCl_3_, 7:3), 105–184 (petrol-CHCl_3_, 5:5), 185–433 (petrol-CHCl_3_ 3:7), 434–579 (petrol-CHCl_3_ 1:9), 580–634 (CHCl_3_), 635–716 (CHCl_3_-Me_2_CO 9:1), 717–778 (CHCl_3_-Me_2_CO 7:3). Frs 394–424 were combined (278.3 mg) applied on a Sephadex LH-20 column (10 g) and eluted with a mixture of MeOH-CHCl_3_ (1:1), wherein 40 subfractions (Sfrs) of 2 mL were collected. Sfrs 15–35 were combined (148.7 mg), applied on a Sephadex LH-20 column (10 g), and eluted with MeOH, wherein 20 sub-subfractions (Ssfrs) of 2 mL were collected (Figure S3). Ssfrs 5-10 were combined (46.0 mg) and crystallized in MeOH to give white crystals of **1** (14.7 mg). Frs 444–453 were combined (312.4 mg) and applied over a column of Sephadex LH-20 (20 g) and eluted with a mixture of MeOH-CHCl_3_ (1:1), wherein 64 subfractions (Sfrs) of 2 mL were collected. Sfrs 38–45 were combined (80.7 mg), applied on a Sephadex LH-20 column (10 g), and eluted with MeOH, wherein 20 sub-subfractions (ssfrs) of 2 mL were collected. Ssfrs 12–17 were combined (21.6 mg) and precipitated in a mixture of CHCl_3_:MeOH; 1:1 to give a white amorphous solid of (**2**) (13.0 mg).

#### 3.2.1. Loliolide (**1**)

White crystal; MP; 173–175 °C. [α]_D_^20′^ -100 (*c* 0.05, CHC1_3_). For ^1^H and ^13^C NMR spectroscopic data (CDCl_3_, 300 and 75 MHz), see Table 1; (+)-HRESIMS *m*/*z* 197.1178 [M+H]^+^ (calc. for C_11_H_17_O_3_^+^, 197.11722).

#### 3.2.2. *epi*-Loliolide (**2**)

White amorphous solid; [α]_D_^20′^ +80 (c 0.05, CHC1_3_); For ^1^H and ^13^C NMR spectroscopic data (CDCl_3_, 300 and 75 MHz), see Table 2; (+)-HRESIMS *m*/*z* 197.1178 [M+H]^+^ (calc. for C_11_H_17_O_3_^+^, 197.11722).

### 3.3. Antibacterial Activity Bioassays

#### 3.3.1. Bacterial Strains and Testing Conditions

Four reference strains obtained from the American Type Culture Collection were included in this study: two Gram-positive (*Staphylococcus aureus* ATCC 29213, *Enterococcus faecalis* ATCC 29212), and two Gram-negative (*Escherichia coli* ATCC 25922, *Pseudomonas aeruginosa* ATCC 27853); as well as one clinical isolate (*E. coli* SA/2, an extended-spectrum β-lactamase producer-ESBL) and two environmental isolates: *S. aureus* 74/24 [43], a methicillin-resistant isolate (MRSA), and *E. faecalis* B3/101 [44], a vancomycin-resistant (VRE) isolate. All bacterial strains were cultured in MH agar (MH- BioKar Diagnostics, Allone, France) and incubated overnight at 37 °C before each assay to obtain fresh cultures. Stock solutions of the compounds were prepared in dimethyl sulfoxide (DMSO—Alfa Aesar, Kandel, Germany), kept at −20 °C, and freshly diluted in the appropriate culture media before each assay. All stock solutions were prepared at a final concentration of 10 mg/mL, and in all experiments, in-test concentrations of DMSO were kept below 1%, as recommended by the Clinical and Laboratory Standards Institute (CLSI) [45].

#### 3.3.2. Antimicrobial Susceptibility Testing

The Kirby-Bauer method was used to screen the antimicrobial activity of the compounds according to CLSI recommendations [46]. Briefly, sterile blank paper discs with 6 mm diameter (Liofilchem, Roseto degli Abruzzi, TE, Italy) were impregnated with 15 µg of each compound, and blank paper discs impregnated with DMSO were used as a negative control. MH inoculated plates were incubated for 18–20 h at 37 °C, and afterwards, the diameter of the inhibition zones was measured in mm.

Minimal inhibitory concentrations (MIC) were determined by the broth microdilution method, as recommended by the CLSI [47]. Two-fold serial dilutions of the compounds were prepared in cation-adjusted Mueller-Hinton broth (CAMHB- Sigma-Aldrich, St. Louis, MO, USA). The tested concentrations ranged from 1 to 64 µg/mL to keep in-test concentrations of DMSO below 1%, avoiding bacterial growth inhibition. Colony-forming unit counts of the inoculum were conducted to ensure that the final inoculum size closely approximated the intended number (5 × 10^5^ CFU/mL). The 96-well U-shaped untreated polystyrene plates were incubated for 16–20 h at 37 °C, and the MIC was determined as the lowest concentration of compound that prevented visible growth. The minimal bactericidal concentration (MBC) was determined by spreading 10 µL of the content of the wells with no visible growth on MH plates. The MBC was determined as the lowest concentration of compound at which no colonies grew after overnight incubation at 37 °C [48]. At least three independent assays were conducted for reference and multidrug-resistant strains.

#### 3.3.3. Antibiotic Synergy Testing

In order to evaluate the combined effect of the compounds tested with clinically relevant antibacterial drugs, the Kirby-Bauer method was used, as previously described [49]. A set of antibiotic discs (Oxoid, Basingstoke, England), to which the isolates were resistant, was selected: cefotaxime (CTX, 30 µg) for *E. coli* SA/2, vancomycin (VAN, 30 µg) for *E. faecalis* B3/101, and oxacillin (OXA, 1 µg) for *S. aureus* 66/1. Antibiotic discs impregnated with 15 µg of each compound were placed on seeded MH plates. The controls used included antibiotic discs alone, blank paper discs impregnated with 15 µg of each compound alone and blank discs impregnated with DMSO. Plates with CTX were incubated for 18–20 h, and plates with VAN and OXA were incubated for 24 h at 37 °C [45]. Potential synergy was considered when the inhibition halo of an antibiotic disc impregnated with a compound was greater than the inhibition halo of the antibiotic or compound-impregnated blank disc alone.

The combined effect of the compounds and clinically relevant antimicrobial drugs was also evaluated by determining the antibiotic MIC in the presence of each compound. Briefly, when it was not possible to determine a MIC value for the test compound, the MIC of CTX (Duchefa Biochemie, Haarlem, The Netherlands), VAN (Oxoid, Basingstoke, England), and OXA (Sigma-Aldrich, St. Louis, MO, USA) for the respective multidrug-resistant strain was determined in the presence of the highest concentration of each compound tested in previous assays (64 µg/mL). The antibiotic tested was serially diluted, whereas the concentration of each compound was kept fixed. Antibiotic MICs were determined as described above. Potential synergy was considered when the antibiotic MIC was lower in the presence of a compound. For compounds **1** and **2**, when it was possible to determine the MIC, the checkerboard method was used instead, as previously described [50]. Fractional inhibitory concentrations (FIC) were calculated as follows: FIC of compound = MIC of compound combined with antibiotic/MIC compound alone, and FIC antibiotic = MIC of antibiotic combined with compound/MIC of antibiotic alone. The FIC index (FICI) was calculated as the sum of each FIC and interpreted as follows: FICI ≤ 0.5, ‘synergy’; 0.5 < FICI ≤ 4, ‘no interaction’; 4 < FICI, ‘antagonism’ [51].

#### 3.3.4. Biofilm Formation Inhibition Assay

The effect of the isolated compounds on biofilm formation was evaluated through the quantification of total biomass using the crystal violet method, as previously described [49,52]. Briefly, the highest concentration of compound tested in the MIC assay was added to bacterial suspensions of 1 × 10^6^ CFU/mL prepared in unsupplemented Tryptone Soy broth (TSB- Biokar Diagnostics, Allone, Beauvais, France) or TSB supplemented with 1% (p/v) glucose (D(+)-Glucose anhydrous for molecular biology, PanReac AppliChem, Barcelona, Spain) for Gram-positive strains. When it was possible to determine a MIC, concentrations ranging between 2 × MIC and ¼ MIC were tested while keeping in-test concentrations of DMSO below 1%. When it was not possible to determine a MIC, the concentration tested was 64 µg/mL. Controls with appropriate concentration of DMSO, as well as a negative control (TSB or TSB+1% glucose alone), were included. Sterile 96-well flat-bottomed untreated polystyrene microtiter plates were used. After a 24 h incubation at 37 °C, the biofilms were heat-fixed for 1 h at 60 °C and stained with 0.5% (*v*/*v*) crystal violet (Química Clínica Aplicada, Amposta, Spain) for 5 min. The stain was resolubilized with 33% (*v*/*v*) acetic acid (Acetic acid 100%, AppliChem, Darmstadt, Germany), and the biofilm biomass was quantified by measuring the absorbance of each sample at 570 nm in a microplate reader (Thermo Scientific Multiskan^®^ FC, Thermo Fisher Scientific, Waltham, MA, USA). The background absorbance (TSB or TSB+1% glucose without inoculum) was subtracted from the absorbance of each sample, and the data are presented as a percentage of control. Three independent assays were performed for reference strains, with triplicates for each experimental condition.

### 3.4. Antioxidant Activity Assays

The antioxidant activity (ABTS, DPPH, and ORAC) of loliolide and *epi*-loliolide were determined. For these determinations, each compound (Section 3.2) was resuspended in distilled water (2 mg/100 μL), and eight successive dilutions were performed (until 0.015625 mg/100 μL). Butylated hydroxytoluene in ethanol (1 mg/100 μL) was used as the control. Three replicates were performed.

#### 3.4.1. The ABTS Method

The ABTS (2,2′-azinobis(3-ethylbenzothiazoline-6-sulphonic acid)) assay was performed according to Gião et al. [53] with some modifications [54]. Shortly, the free radical ABTS was generated through a chemical oxidation reaction with potassium persulfate, with no involvement of an intermediary radical, and its concentration was adjusted with water to an initial absorbance of 0.700 ± 0.020 at 734 nm (Synergy H1, Biotek, Winooski, VT, USA). The sample (20 µL) was allowed to react with 180 µL of the ABTS solution (2,2′-azinobis-(3-ethylbenzothiazoline-6-sulfonic acid) salt, 0.0384 g in 10 mL of ultrapure water mixed with a solution of potassium persulfate, 0.0066 g in 10 mL of ultrapure water) in the dark at room temperature (ca. 25 °C) and the absorbance was read 5 min exactly after in a 96-well microplate (Sarstedt, Numbrecht, Germany). The blank was distilled water (A0). Trolox was used as the standard for the calibration (the standard Trolox calibration curve was prepared at concentrations of Trolox 25–175 µM), and the results were expressed as µmol of Trolox equivalent/100 milligrams of compound (µmol TE/100 mg). Three independent analyses were performed in each triplicate.

#### 3.4.2. The DPPH Method

The DPPH (2,2-diphenyl-1-picrylhydrazyl) assay was carried out according to the procedure described by Alexandre et al. [55] with some modifications [54]. Briefly, a stock solution (600 µM) was prepared by dissolving DPPH (23.6592 mg) in methanol (100 mL), and it was stored at −20 °C in the dark. The working solution (90 µM) was prepared by mixing 15 mL of the stock solution with 85 mL of methanol so that the absorbance reached 0.600 ± 0.100 at 515 nm (Synergy H1, Biotek, Winooski, VT USA). The sample (25 µL) was allowed to react with the DPPH working solution (175 µL) in the dark at room temperature (25 °C) for 30 min in a 96-well microplate (Sarstedt, Numbrecht, Germany). The absorbance was then measured at 515 nm, with distilled water as the blank (A0). Trolox was used as a standard for the calibration (the standard Trolox calibration curve was prepared at concentrations of Trolox 25–175 µM). The results were expressed as µmol of Trolox equivalent/100 milligrams of compound (µmol TE/100 mg). Three independent analyses were performed in each triplicate.

#### 3.4.3. The Oxygen Radical Absorbance Capacity Method (ORAC)

The ORAC assay was performed in a black 96-well microplate (Thermo Scientific, Roskilde, Denmark), following the method described by Dávalos et al. [56] with some modifications [54]. The sample (20 µL) was mixed with 120 µL of fluorescein (FL) solution (final concentration of 70 nM in the well) and 60 µL of AAPH (2,2′-azobis(2-amidinopropane) dihydrochloride), and the mixture was placed in each well. A control with 80 µL of 75 mM phosphate buffer (pH 7.4) and 120 µL of FL was used. A blank of FL and AAPH, using phosphate buffer in place of the antioxidant solution, was also used (Trolox). Eight calibration Trolox solutions (final concentration of 1–8 µM in the well) were used. The mixture was preincubated at 37 °C for 10 min. The AAPH solution (60 µL, final concentration of 12 mM in well) was added rapidly. After immediately placing the microplate in the reader, the fluorescence was recorded at intervals of 1 min for 90 min. A multidetector plate reader (Synergy H1, Biotek, Winooski, VT, USA) with 485 nm excitation and 528 nm emission filters was used. The equipment was controlled by the Gen5 Biotek software version 3.04. AAPH and Trolox solutions were prepared daily, and fluorescein was diluted from a stock solution (1.17 mM) in 75 mM phosphate buffer (pH 7.4). The antioxidant curves (fluorescence versus time) were normalized to the curve of the blank corresponding to the same assay by multiplying the original data by the factor fluorescence blank at t = 0 and dividing by fluorescence control at t = 0. The area under the fluorescence decay curve (AUC) was calculated from the normalized curves. The final AUC values were calculated by subtracting the AUC of the blank from all results. The final ORAC-FL values were obtained using the standard curve (the standard Trolox calibration curve was prepared at concentrations of Trolox 10–80 µM), and the results were expressed as µmol of Trolox equivalent/100 milligrams of compound (µmol TE/100 mg). Three independent analyses were performed in each triplicate.

All results were expressed as mean ± standard deviation of three independent replicates (n = 3). Student’s *t*-test was used to detect significant differences between results on two compounds. The data relative to the three compounds demonstrated normal distribution (Shapiro–Wilk test) and homogeneous variance (Levene test) and were statistically compared using one-way ANOVA, followed by Tukey’s test (*p* ≤ 0.05). SPSS Base 23.0 for Windows (SPSS Inc., Armonk, NY, USA) was used for the statistical analysis.

## 4. Conclusions

Two previously reported monoterpenoids, loliolide (**1**) and *epi*-loliolide (**2**), were isolated from a microalga *Thalassiosira* sp. A4FEXTCA_0018. The structures and absolute configurations of the stereogenic carbons in **1** were confirmed by X-ray analysis, while the absolute configurations of the stereogenic carbons in **2** were confirmed by NOESY correlations. To our knowledge, this is the first report of isolation of loliolide (**1**) and *epi*-loliolide (**2**) from a microalga of the *Thalassiosira* genus. Compounds **1** and **2** did not exhibit either antibacterial or antibiofilm activity. They did not present a remarkable antioxidant activity, especially *epi*-loliolide. Nevertheless, it does not mean that they do not have other interesting biological activities. For example, the antiviral potential of these compounds against various viruses, including but not limited to herpes simplex virus (HSV), human immunodeficiency virus (HIV), or influenza viruses, could be explored in the future. It might also be interesting to investigate the effects of loliolide and/or *epi*-loliolide on the gut microbiota composition and its potential implications for gut health.

## Figures and Tables

**Table 1:** ^1^H and ^13^C NMR (CDCl_3_, 300 MHz and 75 MHz), COSY and HMBC for compound **1**.

Position	δ_C_, Type	δ_H_, (*J* in Hz)	COSY	HMBC
1	-			
2	172.2 C			
3	112.8 CH	5.69, s		C-2, 3a, 4
3a	182.8 C			
4	36.0 C			
5α	47.2 CH_2_	2.00, ddd (14.5, 2.7, 2.7)	H-5β, 6	C-3a, 6, 7, 10
β		1.53, dd (14.5, 3.7)		C-4, 9
6	66.7 CH	4.34, m	H-5α, 5β, 7α, 7β,	
7α	45.6 CH_2_	2.48, ddd (14.0, 2.5, 2.5)	H-6	C-3a, 5, 6, 7a
β		1.77, dd (14.0, 4.0)		C-3a, 6, 8
7a	87.0 C			
8	27.0 CH_3_	1.79, s		C-3a, 7, 7a
9	30.7 CH_3_	1.27, s		C-3a, 4, 5, 6, 10
10	26.5 CH_3_	1.48, s	H-9	C-3a, 4, 5, 9

**Table 2:** ^1^H and ^13^C NMR (CDCl_3_, 300 MHz and 75 MHz), COSY, HMBC and NOESY assignment for compound **2**.

Position	δ_C_, Type	δ_H_, (*J* in Hz)	COSY	HMBC	NOESY
1					
2	171.9, CO				
3	113.0, CH	5.68, s		C-2, 3a, 4, 7a	
3a	181.3, C				
4	35.1, C				
5α	49.7 CH_2_	2.02, ddd (12.8, 4.3, 2.3)	H-5β,	C-3a, 4, 6, 7	
β		1.31, dd (12.2, 12.2)		C-4, 6, 7a, 10	H-5α
6	64.8, CH	4.10, m	H-5α, 5β, 7α, 7β,		H-5α, 7α, 7β, 8, 10
7α	47.8 CH_2_	2.51, ddd (11.8, 4.0, 2.3)	H-6, 7β	C-3a, 5, 6, 7a	
β		1.48, dd (11.8, 11.8)		C-5, 6, 7a, 8	
7a	86.8 C				
8	25.5, CH_3_	1.56, s	H-7β	C-3a, 7, 7a	H-6
9	29.9, CH_3_	1.29, s		C-3a, 4, 5, 10	
10	25.0, CH_3_	1.24, s		C-3a, 4, 5, 9	

**Table 3:** Minimal inhibitory concentration (MIC, in μM) of compounds **1** and **2** against Gram-positive reference and multidrug-resistant strains.

Compound	*E. faecalis* ATCC 29212	*E. faecalis* B3/101 (VRE)	*S. aureus* ATCC 29213	*S. aureus* 66/1 (MRSA)
**1**	>325	>325	>325	>325
**2**	>325	>325	>325	>325

**Table 4:** Antioxidant activity (ABTS, DPPH, ORAC) of compounds **1** and **2**.

Compound	ABTS	DPPH	ORAC
	(µmol TE/100 mg)		
**1**	0.476 ± 0.062 ^b^	0.302 ± 0.019 ^b^	2.786 ± 0.070 ^b^
**2**	n.d.	n.d.	2.520 ± 0.319 ^b^
BHT	65.661 ± 2.993 ^a^	6.482 ± 0.570 ^a^	55.985 ± 3.582 ^a^

Different letters in each column mean statistically significant differences (*p* < 0.05). BHT, butylated hydroxytoluene (control). n.d. not detected.

## Data Availability

Data are contained within the article or Supplementary Material.

## Supplementary Materials

The following supporting information can be downloaded at: https://www.mdpi.com/article/10.3390/molecules29215175/s1; Figure S1-1–6: spectra of compound **1**; Figure S2-1–6: spectra of compound **2**; Figure S3: TLC profile of crude dichloromethane extract of *Thalassiosira* sp.
