# Supplementary material for: Monoterpene Hydroxy Lactones Isolated from Thalassiosira sp. Microalga and Their Antibacterial and Antioxidant Activities
Source: Molecules. 2024 Oct 31;29(21):5175. doi: 10.3390/molecules29215175 (PMC11547300; doi:10.3390/molecules29215175)
Supplement: Supplementary file 1 [file molecules-29-05175-s001.zip › molecules-3259954-supplementary.pdf]

# Supplementary Materials

# Monoterpene Hydroxy Lactones Isolated from *Thalassiosira* sp. Microalga and their Antibacterial and Antioxidant Activities

**Alcina M. M. B. Morais[1], Decha Kumla[1**], Valter F. R. Martins[1], Ana Alves[1], Luis Gales[2,3], Artur M. S. Silva[4], Paulo M. Costa[2,3], Sharad Mistry[5], Anake Kijjoa[2,6], Rui M. S. C. Morais[1*]**

1 Universidade Católica Portuguesa, CBQF - Centro de Biotecnologia e Química Fina – Laboratório Associado, Escola Superior de Biotecnologia, Rua Diogo Botelho 1327, 4169-005 Porto, Portugal; abmorais@porto.ucp.pt (A.M.M.B.M.); decha.ku@go.buu.ac.th (D.K.); anajoao93@hotmail.com (A.A.); rcmorais@porto.ucp.pt (R.M.C.M.)
2 ICBAS—Instituto de Ciências Biomédicas Abel Salazar, Universidade do Porto, Rua de Jorge Viterbo Ferreira, 228, 4050-313 Porto, Portugal; lgales@ibmc.up.pt (L.G.); pmcosta@icbas.up.pt (P.M.C.)
3 Instituto de Biologia Molecular e Celular (i3S-IBMC), Universidade do Porto, Rua de Jorge Viterbo Ferreira, 228, 4050-313 Porto, Portugal
4 Departamento de Química & QOPNA, Universidade de Aveiro, 3810-193 Aveiro, Portugal; artur.silva@ua.pt
5 Department of Chemistry, University of Leicester, University Road, Leicester, LE 7 RH, UK; scm11@leicester.ac.uk
6 Interdisciplinary Centre of Marine and Environmental Research (CIIMAR), Terminal de Cruzeiros do Porto de Leixões, Av. General Norton de Matos s/n, 4450-208, Matosinhos, Portugal

* Correspondence: rcmorais@ucp.pt (R.M.C.M.); Tel.: +351-22-558-0050
** present address: Faculty of Pharmaceutical Sciences, Burapha University, 169 Long Had Bangsaen Rd, Chonburi 20131, Thailand

**Citation:** To be added by editorial staff during production.

Academic Editor: Firstname Lastname

Received: date
Revised: date
Accepted: date
Published: date

**Copyright:** © 2023 by the authors. Submitted for possible open access publication under the terms and conditions of the Creative Commons Attribution (CC BY) license (https://creativecommons.org/licenses/by/4.0/).

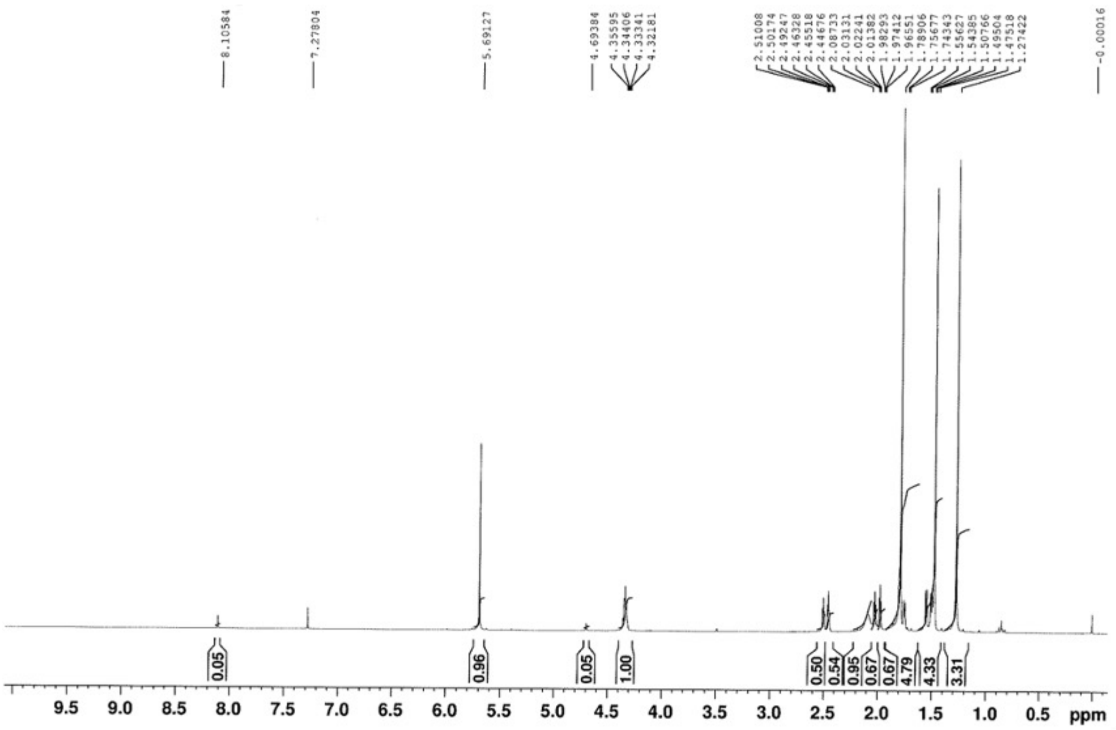


**Figure S1-1**. $^1$H NMR spectrum of compound **1** ($CDCl_3$, 300.13 MHz).

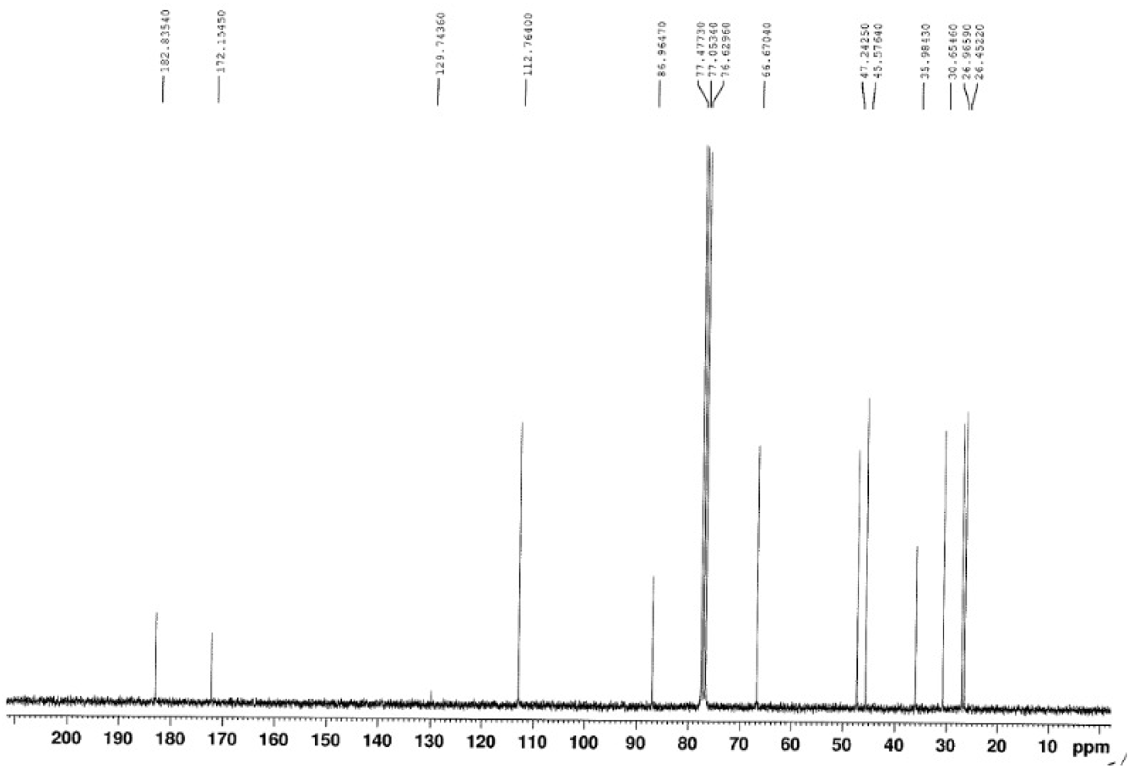


**Figure S1-2**. $^{13}$C-NMR spectrum of compound **1** ($CDCl_3$, 300.13 MHz).

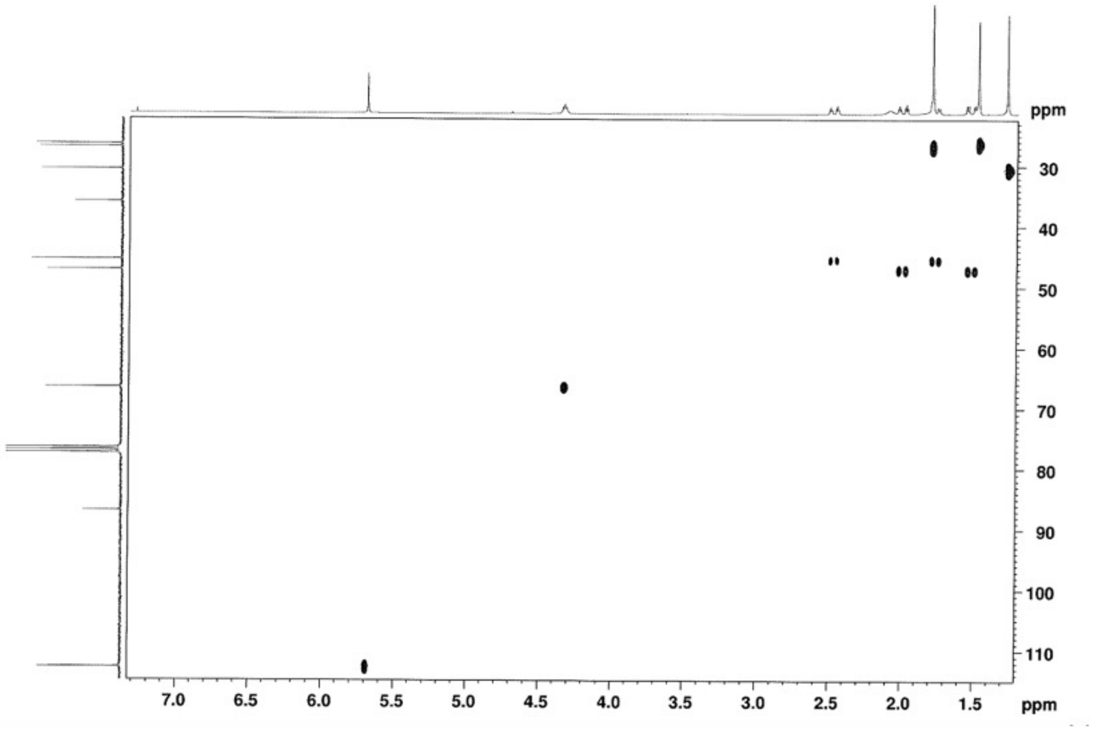


**Figure S1-3**. HSQC spectrum of compound **1** ($CDCl_3$, 300.13 MHz).

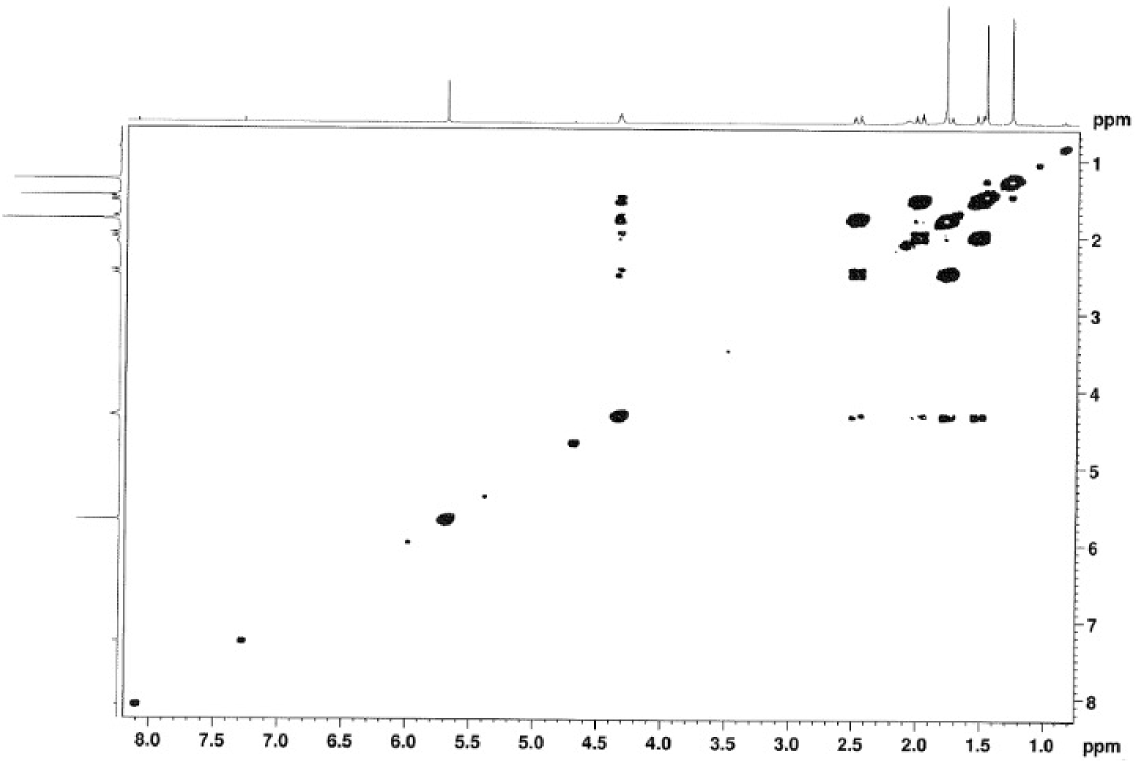


**Figure S1-4**. COSY spectrum of compound **1** ($CDCl_3$, 300.13 MHz).

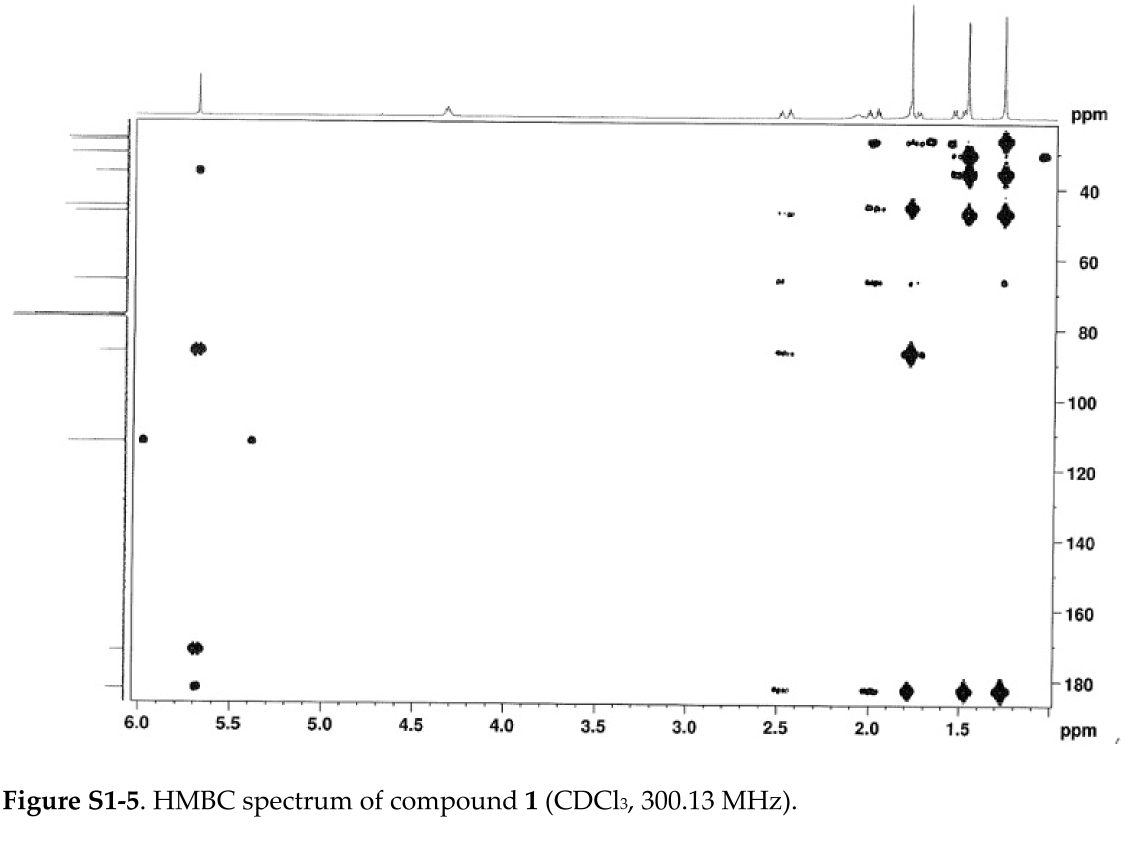


**Figure S1-5**. HMBC spectrum of compound **1** ($CDCl_3$, 300.13 MHz).

Diffraction data were collected at 291 K with a Gemini PX Ultra (Rigaku/Oxford, Neu-Isenburg, Germany) equipped with CuK$\alpha$ radiation ($\lambda$ = 1.54184 Å). The structures were solved by direct methods using SHELXS-97 [1] and refined with SHELXL-97 [1]. Carbon and oxygen atoms were refined anisotropically. Hydrogen atoms were either placed at their idealized positions using

appropriate HFIX instructions in SHELXL, and included in subsequent refinement cycles, or were directly found from difference Fourier maps and were refined freely with isotropic displacement parameters. Full details of the data collection and refinement and tables of atomic coordinates, bond lengths and angles, and torsion angles have been deposited with the Cambridge Crystallographic Data Centre (CCDC).

aktsd394_424

Crystal was monoclinic, space group $P2_1$, cell volume 526.88(6) Å$^3$ and unit cell dimensions $a$ = 6.0774(4) Å, $b$ = 11.6890(7) Å and $c$ = 7.8721(6) Å, and $\beta$ = 109.582(8)° (uncertainties in parentheses). Flack $x$ was refined parameter by means of TWIN and BASF in SHELXL to 0.0(7). The refinement converged to R (all data) = 7.92% and wR2 (all data) = 17.94%. CCDC 2096018.

aktsd115_121

Crystal was monoclinic, space group $C2$, cell volume 5457.2(10) Å$^3$ and unit cell dimensions $a$ = 71.099(12) Å, $b$ = 7.5834(5) Å and $c$ = 10.1218(6) Å, and $\beta$ = 90.419(8)° (uncertainties in parentheses). Flack $x$ was refined parameter by means of TWIN and BASF in SHELXL to 0.2(15). The refinement converged to R (all data) = 18.12% and wR2 (all data) = 43.46%. CCDC 2095981.

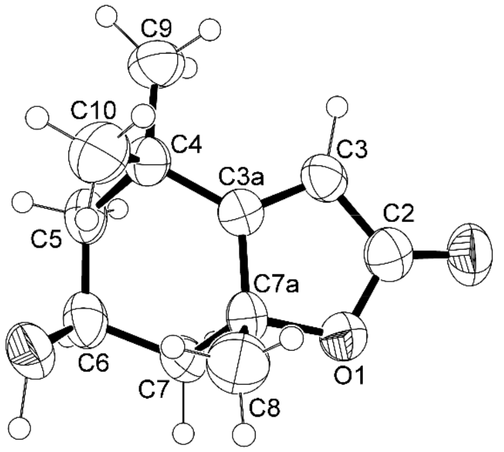


**Figure S1-6**. ORTEP diagram and diffraction data of compound **1.**

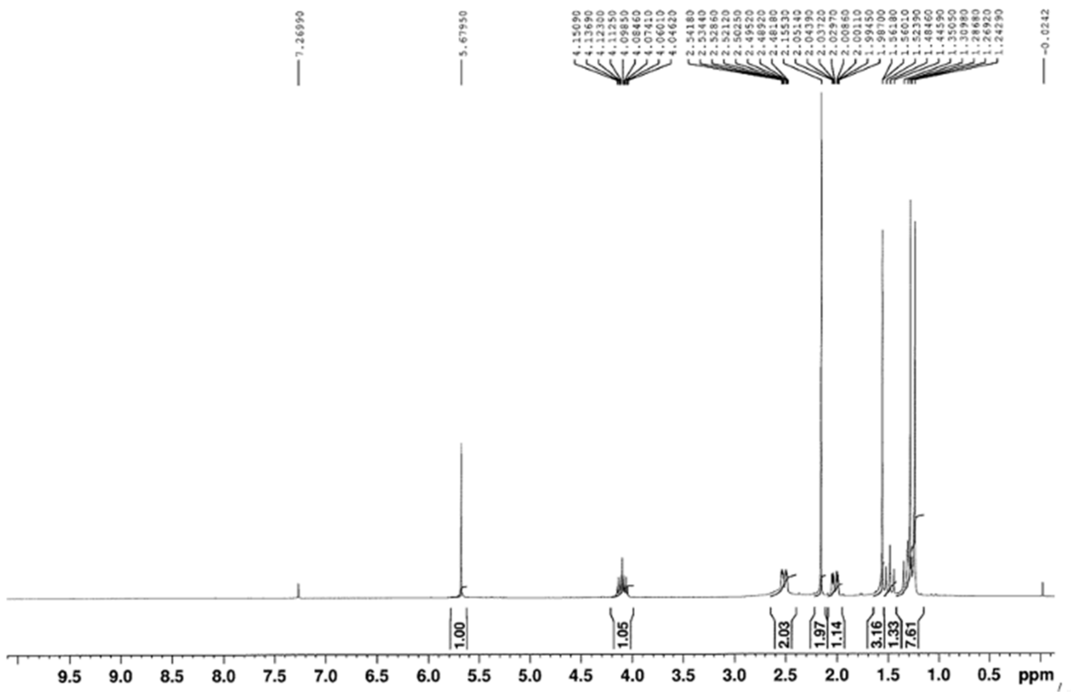


**Figure S2-1.** $^{1}H$ NMR spectrum of compound **2** ($CDCl_3$, 300.13 MHz).

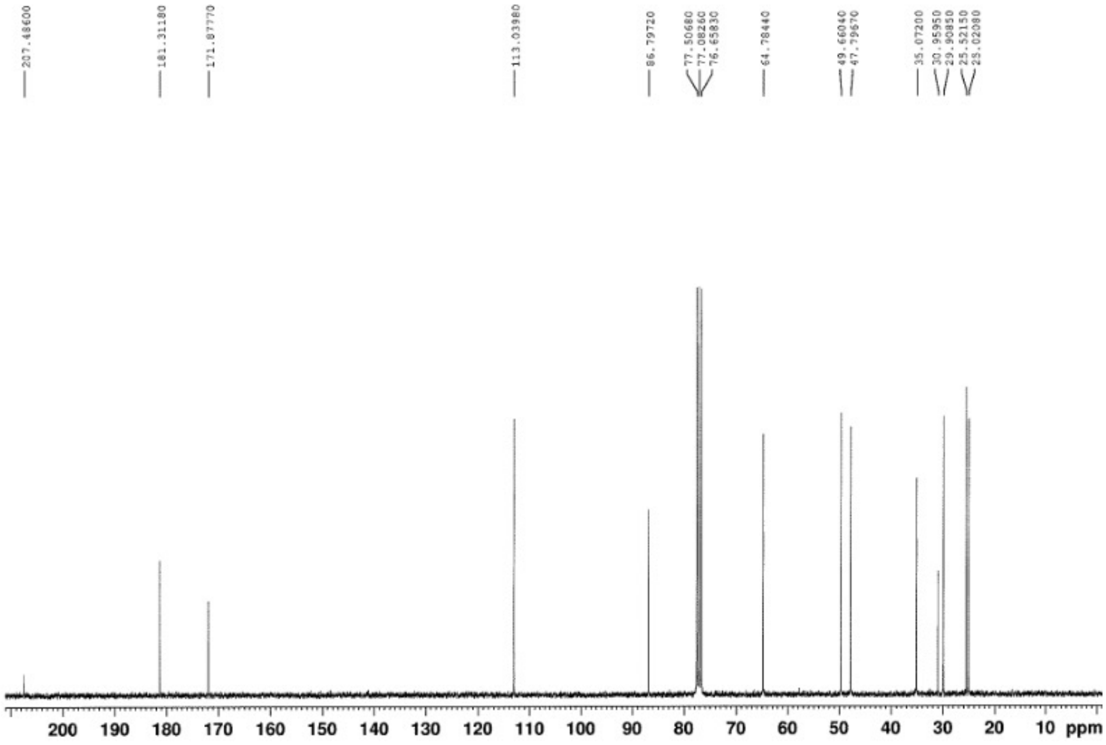


**Figure S2-2**. $^{13}C$-NMR spectrum of compound **2** ($CDCl_3$, 300.13 MHz).

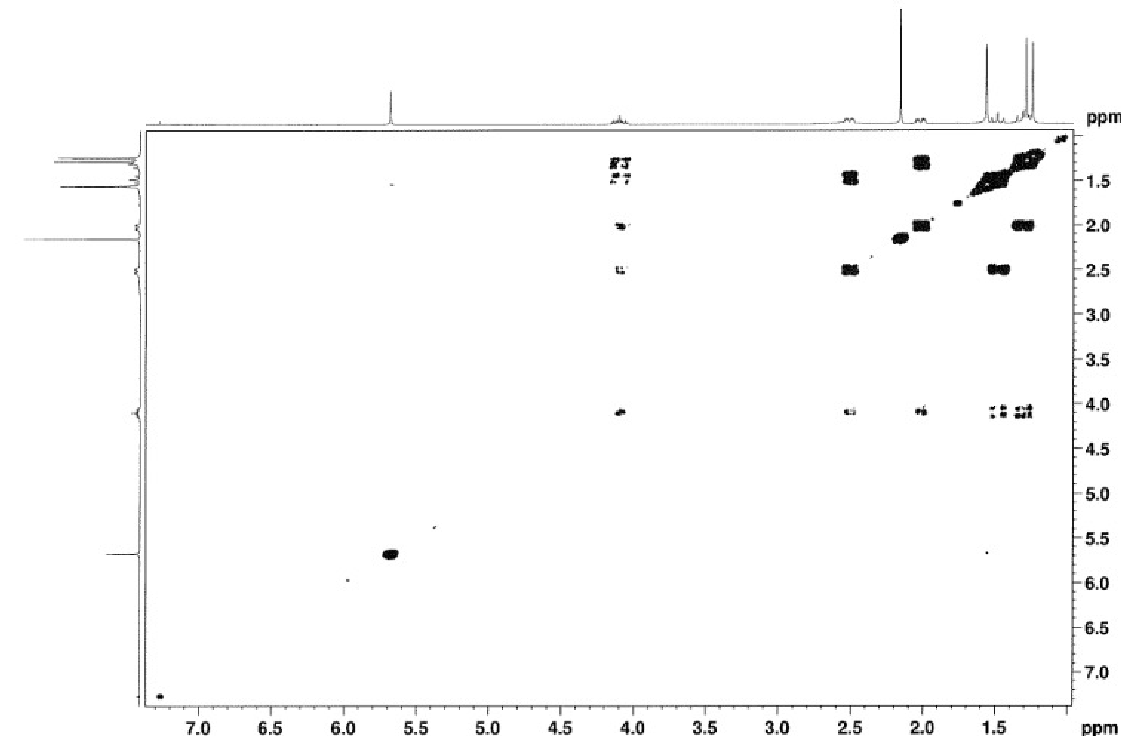


**Figure S2-3.** COSY spectrum of compound **2** ($CDCl_3$, 300.13 MHz).

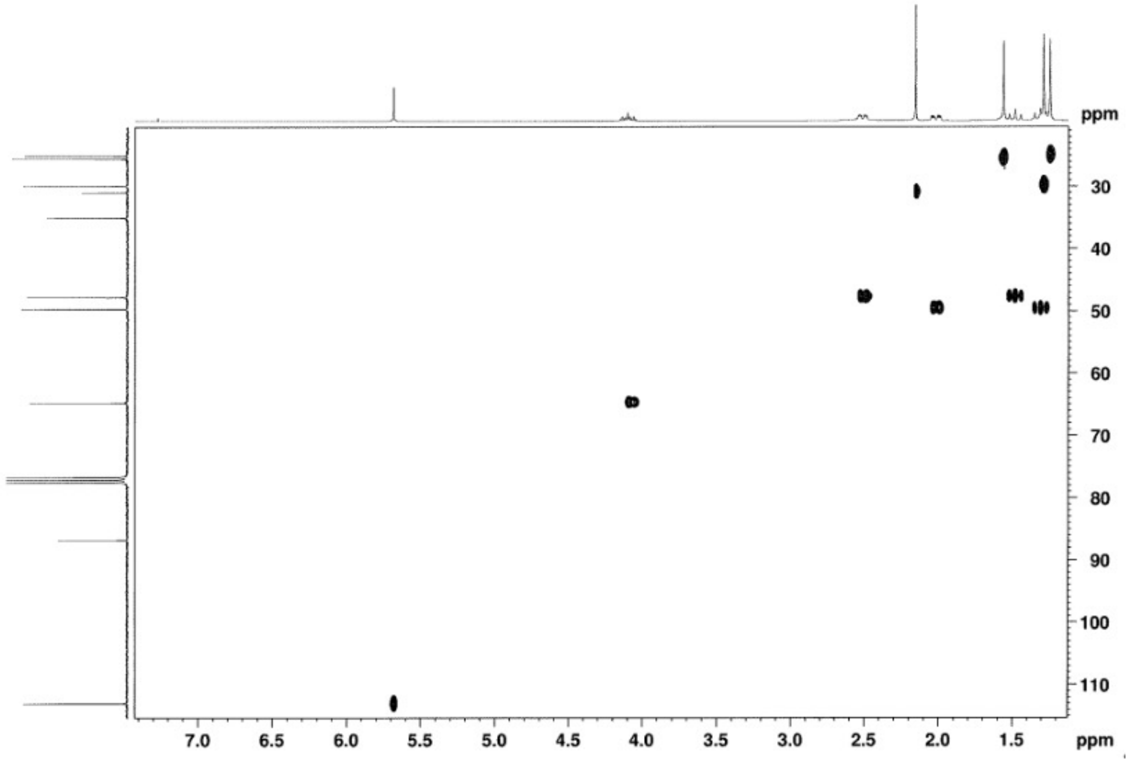


**Figure S2-4**. HSQC spectrum of compound **2** ($CDCl_3$, 300.13 MHz).

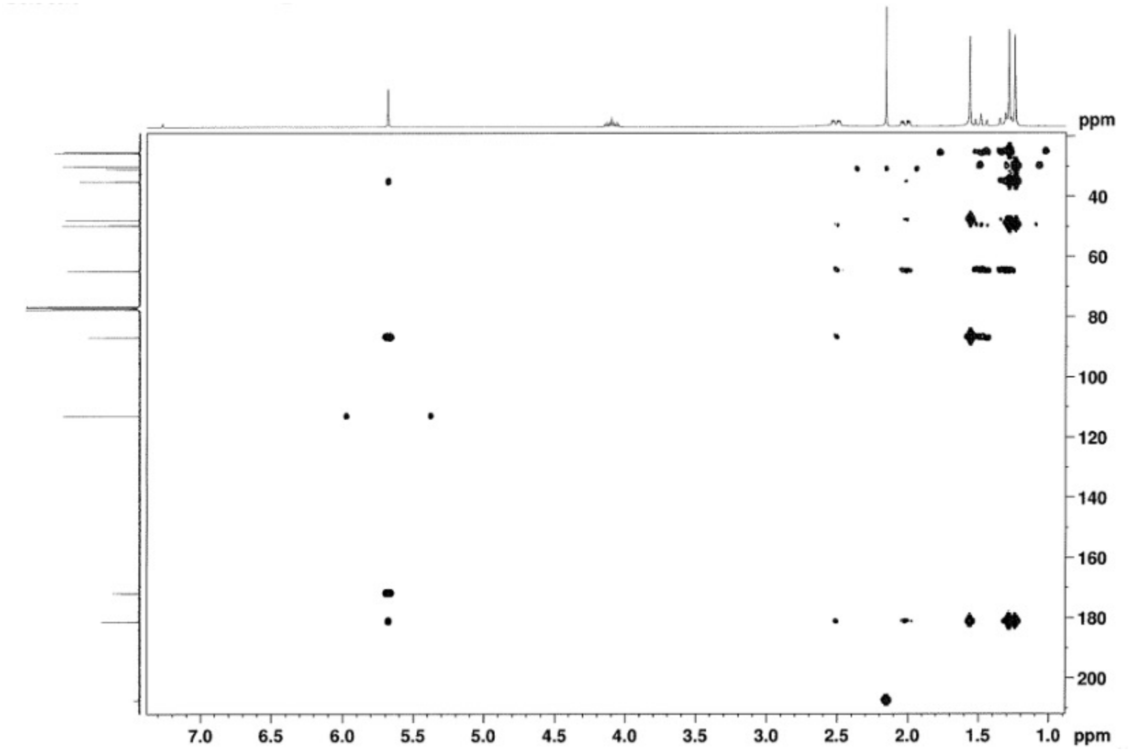


**Figure S2-5**. HMBC spectrum of compound **2** ($CDCl_3$, 300.13 MHz).

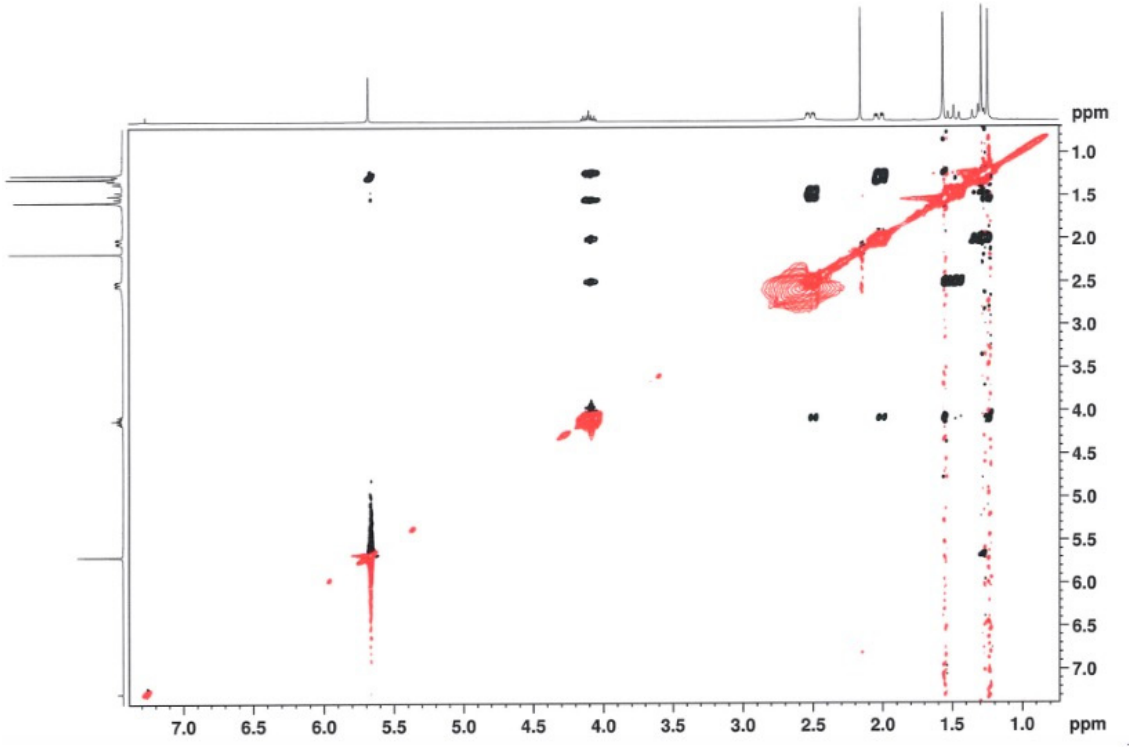


**Figure S2-6**. NOESY spectrum of compound **2** ($CDCl_3$, 300.13 MHz).

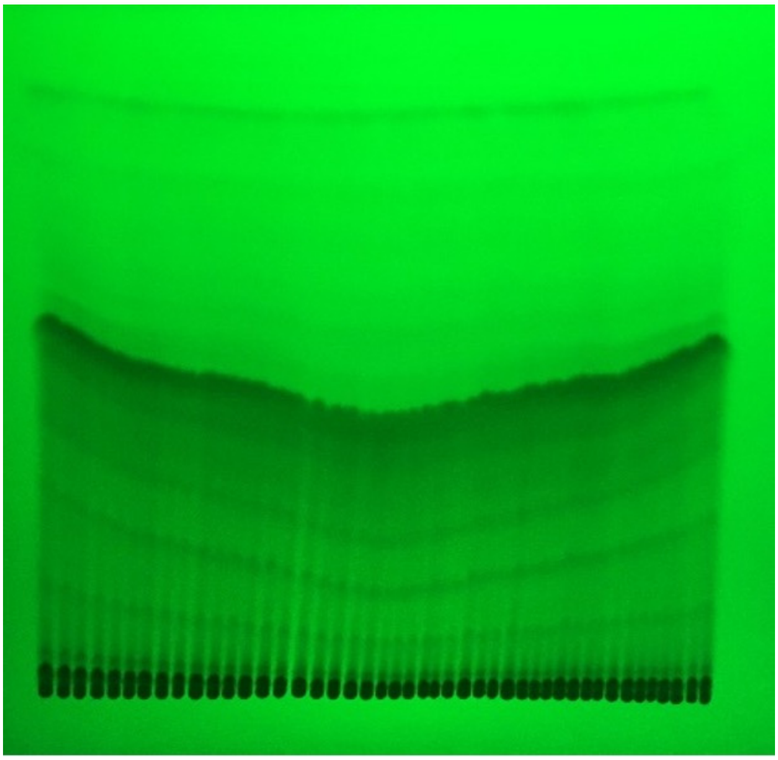

**Figure S3**. TLC profile of crude dichloromethane extract of *Thalassiosira* sp.
